# Can Hypoxic Conditioning Improve Bone Metabolism? A Systematic Review

**DOI:** 10.3390/ijerph16101799

**Published:** 2019-05-21

**Authors:** Marta Camacho-Cardenosa, Alba Camacho-Cardenosa, Rafael Timón, Guillermo Olcina, Pablo Tomas-Carus, Javier Brazo-Sayavera

**Affiliations:** 1 Faculty of Sport Science, University of Extremadura, 10003 Cáceres, Spain; albacc@unex.es (A.C.-C.); rtimon@unex.es (R.T.); golcina@unex.es (G.O.); 2 Departamento de Desporto e Saúde, Escola de Ciência e Tecnologia, Universidade de Évora, 7000-812 Évora, Portugal; ptc@uevora.pt; 3 Comprehensive Health Research Centre (CHRC), University of Évora, 7000-812 Évora, Portugal; 4 Instituto Superior de Educación Física, Universidad de la República, 40000 Rivera, Uruguay; jbsayavera@cur.edu.uy; 5 Polo de Desarrollo Universitario EFISAL, Universidad de la República, 40000 Rivera, Uruguay

**Keywords:** oxygen deprivation, altitude, osteogenesis, bone mineral density, parathyroid hormone

## Abstract

Among other functions, hypoxia-inducible factor plays a critical role in bone–vascular coupling and bone formation. Studies have suggested that hypoxic conditioning could be a potential nonpharmacological strategy for treating skeletal diseases. However, there is no clear consensus regarding the bone metabolism response to hypoxia. Therefore, this review aims to examine the impact of different modes of hypoxia conditioning on bone metabolism. The PubMed and Web of Science databases were searched for experimental studies written in English that investigated the effects of modification of ambient oxygen on bone remodelling parameters of healthy organisms. Thirty-nine studies analysed the effect of sustained or cyclic hypoxia exposure on genetic and protein expression and mineralisation capacity of different cell models; three studies carried out in animal models implemented sustained or cyclic hypoxia; ten studies examined the effect of sustained, intermittent or cyclic hypoxia on bone health and hormonal responses in humans. Different modes of hypoxic conditioning may have different impacts on bone metabolism both in vivo and in vitro. Additional research is necessary to establish the optimal cyclical dose of oxygen concentration and exposure time.

## 1. Introduction

Our understanding of the influence of hypoxia on human physiology has been improved with the discovery of hypoxia-inducible factor (HIF). Low available oxygen leads to the decline of oxygen partial pressure in arterial blood (PiO_2_), which determines HIF stabilisation [1]. HIF translocates to the nucleus where it increases the mRNA expression of a wide variety of genes [2]. Among others, respiratory rate, heart rate and systemic blood pressure are increased to improve oxygen transport and utilisation [3]. If the exposure is maintained for a long period of time, a switch from mitochondrial oxidative phosphorylation to anaerobic glycolysis occurs with concomitant production of reactive oxygen species (ROS) [4]. Whereas higher levels of ROS could be detrimental [5], low levels of ROS can have protective effects on specific cells, tissues and organs [6].

Although hypoxia-driven pathways are vital in normal development and tissue homeostasis, potential mechanisms resulting in osteoblastic activity remain unclear [7]. HIF mediates upregulation of glycolytic enzymes such as pyruvate dehydrogenase kinase 1 (PDK1), lactate dehydrogenase A (LDHA) and glucose transporters (GLUTs) that compensate for the energy inefficiency of glycolysis [8]—the main metabolic pathway during osteoblastic differentiation [9]. Even in the presence of oxygen, bone cells metabolise glucose by a phenomenon known as the “Warburg effect” or “aerobic glycolysis” [10]. Thus, it could be hypothesised that hypoxic exposure could enhance bone formation by promoting glycolysis as the main metabolic pathway. However, HIF also regulates bone remodelling-related genes, such as vascular endothelial growth factor (VEGF), erythropoietin (EPO) and osteoprotegerin (OPG)—the factor that inhibits osteoclastogenesis by counteracting RANKL and therefore, bone reabsorption [11]. Because of the vital role in angiogenic–osteogenic coupling, animal and cell studies have suggested that hypoxic conditioning (HC) could be a potential nonpharmacological strategy for treating hypoxic-ischaemic diseases, including skeletal diseases [12].

HC is a drug-free method based on the adaptation produced by breathing air with low oxygen content [4]. During the last 30 years, HC interventions have enhanced physical and mental functions and the prevention of aging and different diseases in two million patients [13]. HC can be implemented with different patterns and severity, mediating different molecular pathways [14]. An optimal dose in terms of duration, frequency and severity could range from no response at low intensity to a protected state and higher intensities or even a further increase in stimulus could cause negative adaptations [15]. In this sense, some authors have distinguished between different hypoxia modes. “Sustained hypoxia” is characterised by a single episode of hypoxia, which is maintained during a prolonged stay. Hypoxia can also be interrupted by reoxygenation periods and, thus, two modes of hypoxia should be differentiated. “Intermittent hypoxia” is characteristic of obstructive sleep apnoea syndrome (OSAS), where HIF-1 activation is stimulated by many short cycles of severe hypoxia lasting 15–30 s and longer periods of reoxygenation. However, during “cyclical hypoxia”, HIFs accumulate strictly during longer periods of moderate hypoxia (since 12 h), followed by identical reoxygenation periods [14]. Previous reviews have considered the effects of sustained, intermittent and cyclical exposures on cardiovascular and respiratory physiology, health and overall quality of life [1,4,7,13]. It has been shown that “sustained” and “intermittent” hypoxia could lead to deleterious consequences by increasing oxidative stress [16] and producing systemic inflammation [17], whereas “cyclical” hypoxia may lead to a prolonged and sustained state of protection [15]. However, there is currently not a clear consensus regarding the response of bone metabolism to hypoxia [18] and therefore, the optimal dose for HC to achieve beneficial effects is unknown. Due to the lack of systematic reviews on this topic, it will be important to merge the information with the available evidence. Thus, the aim of the present review was to examine the impact of the different modes of HC on bone metabolism. 

## 2. Materials and Methods 

### 2.1. Searches and Article Selection Strategy

This systematic review was carried following Preferred Reporting Item for Systematic Reviews and Meta-Analyses (PRISMA) methodology [19]. The well-known electronic databases selected were Web of Science and PubMed. The selected articles were original articles published between 1900 and 2019. Search terms used: “hypoxi*”, “altitude”, “oxygen deprivation”, ”bone remodelling”, “bone metabolism”, “osteog*”, “bone tissue remodelling”, “bone mineral density”, “growth*”, “pulmonary”, “cancer” and “tumour”. The exact search strategy can be found in Appendix A. The search was finalised on the 1 March 2019. The main author deleted duplicate articles. 

Inclusion criteria that were defined for article selection: (1) written in English; (2) experimental study on bone remodelling parameters; (3) containing a sample of healthy organisms; and (4) application of a hypoxic treatment (modification of ambient oxygen). Studies were excluded if they were only presented once as a conference, congress or seminar. These criteria were evaluated first by the titles and abstracts by two authors (A.C.-C. and M.C.-C.) independently. If they met the inclusion criteria or if the title and abstract did not provide sufficient information, full articles of these studies were obtained to apply the criteria at full text by the same two authors. To resolve any data discrepancies, group discussions were conducted until a mutual consensus was reached. A third expert (J.B.-S.) was consulted when a consensus could not be reached. 

### 2.2. Risk of Bias

The internal quality of each study was assessed using the Office of Health Assessment and Translation (OHAT) Risk of Bias Rating Tool [20]. Two review authors (M.C.-C. and A.C.-C.) independently assessed 11 risk of bias questions using the 4-point scale ranging from low to high risk of bias options. Discrepancies between reviewers were resolved using consensus. For both in vivo and in vitro studies, an initial confidence rating was given, which was subsequently downgraded or upgraded according to factors that decrease or increase confidence in the results [21,22].

To establish a credible link between hypoxic exposure and bone health effect, confidence ratings were assigned to individual study designs and translated into a level of evidence (“high”, “moderate”, “low”, “evidence of no health effect” and “inadequate evidence”). 

### 2.3. Data Extraction

One author (M.C.-C.) applied the data extraction. Another author (J.B.-S.) verified this extraction. Details were extracted regarding (a) sample: type (i.e., human, animal or cell), age and sample size; (b) study design: conditions, exposure type or timing (i.e., normobaric or hypobaric; expansion, proliferation or differentiation); protocol (i.e., sustained, cyclic or intermittent) and duration, frequency and hypoxic level; and (c) effects of hypoxia on bone outcomes.

## 3. Results

### 3.1. Article Selection

**Figure 1:**
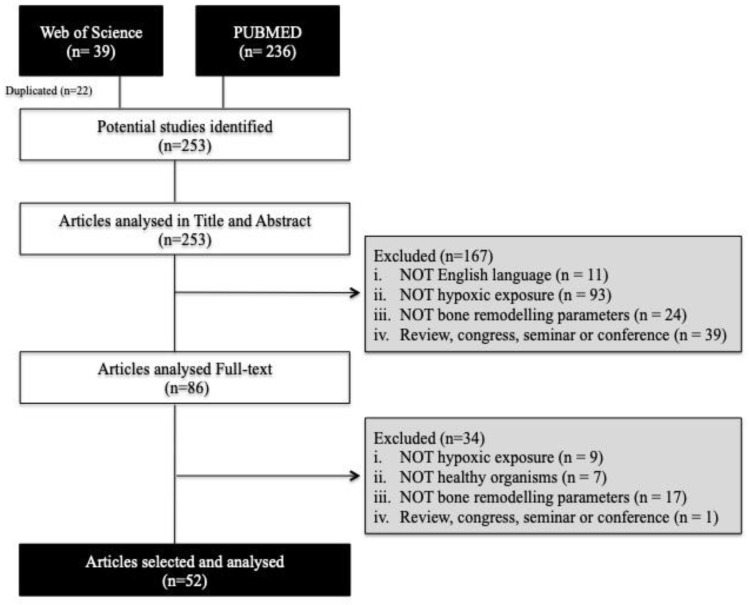
Flowchart of article searches and selection strategies.

The initial electronic database search resulted in a total of 39 citations in Web of Science and 236 in PubMed. After deleting 22 duplicates, 253 studies were analysed by title and abstract by applying the inclusion and exclusion criteria. A total of 167 studies were excluded and 86 potentially relevant studies were selected for full-text review. Fifty-two studies were identified in our systematic search from which risk of bias was described and data extracted. A flowchart of the search procedure can be found in Figure 1. 

### 3.2. Risk of Bias

Results from the risk of bias assessment are shown in Appendix B and Appendix C. Evidence summarised from animal, human and in vitro studies suggested a range of likely high to definitely low level of confidence (see Appendix D). Selective reporting was considered the most pertinent domain, rating from likely low and definitely low for animal and human in vitro studies. Conversely, performance domain was identified at likely high or low level of confidence. This domain was likely high for all human studies.

In the association between hypoxic exposure and bone health effect (see Appendix E), moderate confidence ratings were assigned to epidemiological studies (human cohort and cross-sectional) whereas high confidence was established in experimental studies (in vitro, animal or human).

### 3.3. Data Extraction

Table 1 and Table 2 summarise the data from 39 in vitro and 13 in vivo studies. Table 1 shows the results of the extraction data of the in vitro studies. Experimental details from the in vivo studies can be found in Table 2.

#### 3.3.1. In Vitro Studies

Thirty-nine studies analysed the effect of sustained or cyclic hypoxia exposure on bone remodelling parameters in different cell models from animals (14 studies) or humans (34 studies). Most commonly used stem cells models were: bone marrow stromal cells (BMSCs; *n* = 26), adipose-derived stromal cells (ASCs; *n* = 8), placental mesenchymal stem cells (pMSCs; *n* = 1), tendon-derived stem cells (TDSCs; *n* = 1), umbilical cord perivascular cells (UCPVCs; *n* = 1) and cartilage endplate stem cells (CESCs; *n* = 1). Only three of the in vitro studies reviewed exposed osteoblasts or osteocytes to sustained hypoxia [30,47,53]. Thirty-two studies included in this review applied sustained hypoxia protocols. Sustained hypoxia was administered from one to 28 days throughout the different timelines of cellular differentiation (expansion, *n* = 16; proliferation, *n* = 1; and differentiation, *n* = 27). Related to the hypoxia level, the dose ranged from 0.1 to 7% PiO_2_. Severe hypoxia (<3% PiO_2_) was applied more often (*n* = 33) than moderate hypoxia (>3% PiO_2_; *n* = 11). Only one study applied cyclic hypoxia (two bouts of 3 min per day) over the course of 15 days at 1, 3, 5 and 10% PiO_2_ in human BMSCs [58]. Runt-related transcription factor 2 (RUNX2; *n* = 15), alkaline phosphatase (ALP; *n* = 11), collagen type 1 alpha 1 (Col1A1; *n* = 6), osteopontin (OPN; *n* = 6) and osteocalcin (OC; *n* = 11) were the most studied genes. In terms of protein expression, RUNX2 (*n* = 5) and ALP (*n* = 14) were the predominant parameters. Mineralisation capacity was assessed using ALP activity (*n* = 17) and calcium deposits (*n* = 23).

#### 3.3.2. In Vivo Studies

Three studies carried out in animal models implemented two different types of HC: sustained and cyclic hypoxia. All interventions involved daily exposure with a hypoxic level between 3000 and 6000 m over a prolonged period from 2 to 5 weeks [63,64,72]. Cyclic protocols adopted a pattern of exposure to hypoxia followed by the same time course of exposure to normoxia, lasting 4 [63] and 5 h [64] per day. Six out of the ten studies in humans, included in this review, analysed the effect of sustained or cyclic hypoxia in healthy active [60,61,62,65,66] or sedentary adults [67]. Three studies [60,61,62] implemented sustained HC corresponding to periods of 16, 24 and 60 weeks. The hypoxic level during these stays ranged from 2500 (moderate level) to 6700 m (severe level). Whereas Martinez-Guardado, (2019) and Ramos-Campos, (2015) [65,66] studied the effects after 7 and 8 weeks, respectively, of normobaric cyclic hypoxia training at 15% PiO_2_ (2 days per week; 60 min per session) and [67] applied bed rest or ambulatory normobaric hypoxia during 21 days at 4000 m of simulated altitude. Finally, four observational human studies analysed the effect of OSAS level (namely intermittent hypoxia) on bone remodelling parameters [68,69,70,71]. Healthy bone was evaluated with different parameters: bone volume (BV/TV; *n* = 2); trabecular number (Tb.N.; *n* = 2); bone mineral density-total (BMD-total; *n* = 6); bone mineral density-spine (BMD-spine; *n* = 3); speed of sound (SOS; *n* = 2) values of the radius, metatarsal and phalanx; and T-score of the radius and phalanx (*n* = 2). Additionally, bone formation and resorption markers as well as bone specific alkaline phosphatase (BAP; *n* = 2), ALP (*n* = 2), 25-hydroxy vitamin D3 (25-Vit D; *n* = 2), intact parathyroid hormone (i-PTH; *n* = 2), C-terminal propeptide of type I collagen (CICP; n = 2), N-telopeptide of type I collagen (NTX; *n* = 2), C-terminal telopeptide (CTX; *n* = 2), urinary DPD (*n* = 2), creatine ratio (DPD/Cr; *n* = 2) and OPG (*n* = 1) were analysed to determine the effects of different HC modes.

## 4. Discussion

The present review examined the impact of the different HC modes on bone metabolism. Overall, disparity in protocols, MSC sources and the composition of the cultive media [31,40] used has made it difficult to establish the role of HC in osteogenic differentiation [31]. Conversely, HC modes might have a different effect on skeletal health of animals and humans. A sustained hypoxic environment could negatively influence bone mass and bone quality when tissue PiO_2_ falls below 40 mmHg. However, short episodes with modest levels of hypoxia (9–16% PiO_2_) could lead to benefits [7] if it is administered repeatedly [73]. Finally, intermittent hypoxia (associated with OSAS) may have unfavourable effects on bone metabolism [69] and other organs and systems [74,75]. 

### 4.1. In Vitro Studies

#### 4.1.1. Sustained Exposure

To evaluate the effects of hypoxia on osteogenic differentiation, the genetic and protein expression of different biomarkers were evaluated. Sustained hypoxia was administered in MSCs derived from bone marrow, adipose tissue, placental, tendon, umbilical cord or cartilage endplate from one to 28 days throughout the different timelines (expansion, proliferation or differentiation).

While some studies showed decreases in RUNX2 gene expression [23,27,42,44,45,47,49,50,53,57,72], expression was maintained in three studies [31,34,36]. A shorter exposure (3–14 days), applied in two studies, increased RUNX2 gene expression [32,34]. RUNX2 protein expression decreased in the five reviewed studies [27,39,48,49,53], which applied 5–21 days of sustained hypoxia at 1–2% PiO_2_. Overall, data from mRNA expression analysis could differ from protein expression by the post-translational modification of osteogenic biomarkers that may be a critical step dependent on the duration of hypoxia [26]. Osteogenic differentiation is controlled by RUNX2—a specific transcription factor that can promote or inhibit the expression of osteogenic differentiation-related genes [33]. Expression of RUNX2 can induce the synthesis of early (e.g., ALP and COL1A1) and late markers of osteoblast differentiation (e.g., OC) [31]. Thus, sustained hypoxic exposure of 1–5% PiO_2_ for 2–28 days show contradictory results and so how this exposition affects to the osteoblastic differentiation cannot establish. 

Sustained hypoxia protocols of 1–2% PiO_2_ for 5–21 days showed lower ALP gene expression [27,53,72,76], while moderate hypoxia (2–5% PiO2) for 3–14 days showed greater expression [33,34,35]. Protein expression of ALP increased when lower severity (>2% PiO_2_) and length of exposure (2–21 days) were applied [26,32,34,55,76,77,78]. Finally, ALP activity decreased in 11 of 18 studies after severe hypoxia (1–2% PiO_2_) was applied for 2–28 days [23,31,42,45,47,49,52,53,54,57,77]. ALP is a biomarker of bone growth and development as it produces an alkaline environment allowing calcium to crystallise and strength to be achieved [27]. Thus, it seems that exposures with greater severity (1–2%) and longer duration (up to 28 days) could negatively affect ALP; however, protein expression increased when moderate hypoxia (<2%) was applied for a shorter period of time (up to 21 days).

Similar to other genes, Col1A1 expression showed contradictory results. While expression of the Col1A1 gene was maintained at 5% PiO_2_ [35,36], it decreased with 2% PiO_2_ for 7 [53] or 21 days [23], but increased with 2% PiO_2_ for 12 days [33]. Col1A1 is an indicator of the efficiency of the final osteogenic potential [79]. Therefore, a lower expression of COL1A1 would indicate a decreased efficiency in osteogenic potential [32]. In this sense, moderate oxygen concentration (2–5%) with a moderate exposure time may promote bone formation. 

During bone formation, late in the mineralisation process, noncollagenous glycoproteins such as OPN and OC are abundant in the bone matrix with biological and mechanical functions of bone [80]. Decreases in OPN expression activate osteoclastic bone resorption and inhibit osteoblastic bone formation [81]. A lower genetic expression of OPN was observed following severe hypoxia protocols (1–2% PiO_2_) for 21 days [27,40,45,46,49], but OPN expression was maintained with 5% PiO_2_ during cellular expansion and differentiation of human ASCs [42]. Nine of the eleven studies that evaluated this parameter showed a lower expression of the OC gene when severe hypoxia protocols of 5–28 days were applied [27,40,45,46,49,72,76]. Only two studies [33,35] showed increased expression with a more moderate dose (>2%) and lower exposure time (3–12 days). 

Finally, in the present review, calcium deposits maintained the same values compared with the normoxia groups in four studies, after low oxygen availability between 1% to 5% PiO_2_ was applied for 14–21 days [33,37,38,41]. In BMSCs, the most severe doses (<2%) showed decreased calcium deposits [27,29,40,41,46,49,51,52,76]. Previous researchers have suggested that there may exist a basal threshold of tissue oxygenation that regulates the deposition of minerals in the extracellular matrix [53]. Thus, similar to osteocytes buried in mineralised bone, lower PiO_2_ may result in low ALP activity and minimal mineralisation potential [53]. 

Overall, it is difficult to clarify the role of sustained HC on osteogenic differentiation [31]. The differences in reported effects on cellular behaviours may be due to disparity in protocols, MSC sources from different species or tissues and/or the composition of the cultive media [31,40].

Different effects have been reported due to discrepancies in oxygen concentration and exposure time. It is notable that 2% may represent a critical concentration, and therefore oxygen concentrations above 2% could promote osteogenic responses [31]. However, long-term or chronic exposure to hypoxia was reported to inhibit osteogenic differentiation. Effects of hypoxia on osteogenic differentiation may be time-dependent: osteogenesis could be accelerated in the early period, but sustained long-term hypoxia could result in poor osteogenesis [27]. On the other hand, previous studies have reported that under low O_2_ conditions, MSCs proliferate faster and for a longer period of time [29]. However, maintaining the exposure during cellular differentiation could maintain the undifferentiated characteristics of these cells [40]. Conversely, subsequent inductions under normoxic conditions during differentiation could maintain or improve the differentiation potential [82]. Thus, the timing of the exposure of MSCs to hypoxia could be important in osteogenic differentiation.

According to the origins of different tissues, MSCs show altered differentiation in response to hypoxia [48]. Osteogenesis may only be induced in periodontal ligament MSCs [83] under sustained hypoxic conditions but inhibited in bone marrow and adipose MSCs [50,57,84,85].

#### 4.1.2. Cyclic Exposure 

In a study conducted by [58], the effects of 15 days of different doses (1, 3, 5 and 10% PiO_2_) of cyclic hypoxia (two bouts of 3 min per day) during the differentiation of human BMSCs were studied. Compared with normoxia conditions, low oxygen concentration increased cell proliferation (especially at 3% PiO_2_) but inhibited osteoblastic differentiation by decreasing RUNX2 and OC gene expression. Thus, the exposure time used was not sufficient to promote osteogenic differentiation.

### 4.2. In Vivo Studies

#### 4.2.1. Sustained Exposure

Some reports have indicated that residency at altitude may cause a marked deterioration in different indices of skeletal health [61]. The studies included in this review, which measured different indices of skeletal health in animals and humans, reported similar findings. At extreme altitude, healthy rats showed a decrement in the BV/TV, Tb.N. and BMD-total after 3 weeks of sustained simulated hypoxia at 6000 m [59]. In addition, 14 healthy young adults were exposed to bed rest or ambulatory normobaric hypoxia for 21 days at 4000 m of simulated altitude [67]. Bone mineral content-total (BMC-total) decreased after bed rest protocols and increased after ambulatory hypoxic conditions. In addition, at high altitude, a group of five healthy active male adults [60], who participated in an expedition of 24 weeks at 2500 m of altitude showed a decrease in BMD-spine. Also, the Indian army composed of healthy males stayed at high altitude (3450 m) for 16 weeks [62] and showed a decrease in bone strength (SOS values of the radius, metatarsal and phalanx, and T-score of the radius and phalanx). Thus, a sustained hypoxic environment could negatively influence the bone mass and bone quality when tissue PiO_2_ falls below 40 mmHg. HIF could affect the activity of multiple skeletogenic cells involved in angiogenesis, extracellular matrix formation and resistance to infection [59,86]. Nevertheless, hypoxic conditions could enhance the differentiation of osteoclasts [87] and modulate their binding to resorption sites [88]. 

In addition to skeletal health, prolonged residency in a hypoxic environment is associated with changes in turnover of bone metabolism coupled with specific endocrine adaptations. Bone formation markers such as ALP, bone-specific alkaline phosphatase (B-ALP), 25-Vit D, parathyroid hormone (PTH) and resorption markers (e.g., carboxy-terminal collagen cross-link (CTX), urinary DPD and creatine ratio (DPD/Cr)) were evaluated after a stay at extreme and high altitudes [61,62]. The study reported that after 4 months at extreme altitude, ALP, B-ALP and CTX decreased and the DPD/Cr ratio did not show any significant change. These results indicate activation of the bone resorption process at extreme altitude. The DPD/CR ratio, B-ALP, protein released by osteoblasts and CTX were lower at high altitude. Decreased formation and expression of bone resorption markers reflected a lower bone turnover at high altitude.

PTH is the major hormone regulating calcium metabolism [89]; this hormone aids in the production of bone-destroying osteoclasts and consequently speeds up bone remodelling and the release of Ca and other minerals in usable forms [90]. Whereas PTH levels are increased at extreme altitude and decreased at high altitude, serum 25-Vit D showed a significant decline at both high and extreme altitudes. Decline of 25-Vit D remains speculative but may be due to declined conversion of 25(OH) to 1.25(OH) D3 under conditions of low oxygen [61]. As a result, increased PTH may be required to increase this conversion to stimulate intestinal absorption of calcium. Calcium levels are maintained at extreme altitude and significantly increased at high altitude. These studies suggest that sustained hypoxia is associated with a decline in bone turnover due to reduced formation and expression of bone resorption markers. Whether this decline in bone turnover can lead to an increase in calcium deposition in bones during residency at high altitude remains to be determined [61]. At extreme altitudes, more significant changes occur in hormonal and biochemical bone remodelling parameters.

Compared to in vitro studies, in vivo environments are much more complex, and more factors related to the hypoxic environment may be responsible for the impaired bone strength and quality [59]. Weight loss [91], increased lean mass [65], lowered basal metabolic rate [59], decreased activity levels [92], insufficient vitamin D levels [60] or dietary changes in Ca^2+^ [93], vitamin C [94] or vitamin D [95] can also influence the BMD, and thus play an important role in healthy bone.

#### 4.2.2. Cyclic Exposure

Different findings have been reported following the application of cyclic HC protocols in humans and animals. Increases in the BMD-spine of rats were observed after 5 weeks of cyclic normobaric hypoxia (5 days per week; 5 h per day) at 4500 m [64]. In addition, healthy active adults showed improved BMD-total after 8 weeks of normobaric hypoxic training [65]; however, in trained triathletes [66], 7 weeks of normobaric cyclic hypoxia training at 15% PiO_2_ (2 days per week; 60 min per session) resulted in no reported changes in BMD-total. In another study, cyclic HC exposure was applied for 2 weeks, 4 h per day at 3000 to 5000 m maintaining BV/TV, Tb.N., BMD-total and BMC-total level in healthy rats [63]. The variations in the present findings may be partly explained based on previous findings that explain how the numbers of hypoxic episodes, severity and duration of total exposure may result in different physiological responses [13]. In this sense, a small number of short episodes with modest levels of hypoxia (9–16% PiO_2_) could lead to benefits [7] administered repeatedly over days or weeks [73]. 

Arterial hypoxemia has been postulated to cause systemic inflammation by activation of regulatory pathways and cytokines, thus causing bone loss (see in vivo studies; Intermittent Exposure) [96]. However, rats exposed to cyclic normobaric hypoxia for 5 weeks showed higher BMD [64]. Increased ROS production will activate proinflammatory cytokines, which cause production of nitric oxide (NO) in osteoblasts and osteoclasts, among other cells. It is known that NO regulates osteoclast-mediated bone reabsorption, activating osteoblastic activity and inhibiting RANKL expression [8,97]. Inhibition of NO in these studies showed BMD levels significantly elevated as well, indicating that there are further mechanism(s) besides the NO-mediated effect in increasing BMD following cyclic hypoxic exposure, such as increased oxidative stress or a VEGF-mediated effect [64]. On the other hand, no significant changes in the level of Ca, P and PTH following hypoxic exposure could indicate a restrain of osteoclastic activity and/or stimulation of osteoblastic activity, affecting bone metabolism via multiple mechanisms. Overall, cyclic modes may inhibit osteoclastic activity and/or stimulate osteoblastic activity; more research is needed to understand these mechanisms [98]. 

Hormonal factors could also have an influence on the achieved effects. A group of ovariectomised rats were exposed to the same dose of hypoxia showing a decrease in the assessed outcomes [63]. This suggests that imbalanced bone remodelling caused by hypoxia occurs in female rats when oestrogen is deficient, leading to possible accelerated bone loss in postmenopausal women [63]. Although long-term exposure to cyclic moderate hypoxia could have benefits without detrimental effects, establishing the optimal cyclical dose in terms of episode duration and time of exposure for the treatment of skeletal diseases with low oxygen concentrations requires substantial additional research [73].

#### 4.2.3. Intermittent Exposure

Nocturnal breathing difficulty, specially, sleep apnea, results in intermittently low oxygen levels by reductions of airflow while sleeping [99]. While some reports have studied the relationship between sleep apnea and bone health, it remains unclear. In the present review, observational human studies, which analyzed the effect of OSAS level on bone remodelling parameters [68], observed similar values in BMD-spine [70] and higher in BMD-total values compared with healthy adults. These findings could be explained by osteogenesis–angiogenesis coupling phenomenon, induced by HIF secretion [68]. HIF may promote osteogenic factors via VEGF or ALP expression [68]. However, gender, comorbidities, ethnic group or age could lead conflicting results. Usual comorbidities, characteristics of OSA patients, could affect to bone health and healthy controls could not be considered as a valid control. In this sense, others studies included in this review such as intermittent hypoxic expositions, which excluded OSA patients with comorbidities, showed lower BMD in femoral neck [69]. In addition, higher values of bone resorption markers (e.g., CTX) and similar OPG values were found in subjects with severe OSAS [69,71]. A previous review showed that OSAS’s patients had increased circulating markers of systemic inflammation, which may contribute to the development of osteopenia [96]. Chronic hypoxia could reduce the expression of bone formation markers such as B-ALP or type I collagen [88], and promote the function of osteoclasts by increasing cytokines such as interleukin-6 [88,100]. 

While some studies have shown that intermittent hypoxia may have a protective role in bone health [68], other studies with larger samples suggest that OSAS may have unfavourable effects on bone metabolism [69]. In addition, intermittent hypoxia, which is associated with OSAS, causes an increase in oxidative stress with negative effects on other organs and systems [74,75]. 

## 5. Conclusions

In conclusion, different modes of HC may lead to different impacts on bone metabolism in both in vivo and in vitro models. While sustained and intermittent hypoxia might inhibit osteogenic differentiation and promote osteoclast function, cyclical hypoxia has been presented as a promising strategy to beneficially impact bone metabolism. In this sense, moderate oxygen concentration (above 2% in vitro and 9–16% in vivo) administered repeatedly over days or weeks may promote mineralisation potential, inhibit osteoclast activity and/or stimulate osteoblast activity. However, additional research is necessary to establish the optimal cyclical dose in terms of oxygen concentration and exposure time (episode duration, number of exposures per day and length).

## Figures and Tables

**Table 1:** Experimental details of in vitro studies included in this review.

Hypoxia Effects on Outcomes	Hypoxia Level (% PiO2)	Duration, Frequency	References	Confidence Rating
**Sustained Exposure**				
**↑**RUNX2-g **↑**ALP-g **↑**ALP **↑**ALP-activity **↑**Col1A1-g **↑**Col1A1 **↑**Osteocalcin-g **↑**Osteocalcin **↑**Calcium deposit	0.1%	1 days	Huang, 2012 [23]	Moderate
	1%	2 days	Kalinina, 2015 [24]	Moderate
		12 days	Deschepper, 2011 [25]	Moderate
		21 days	Gao, 2013 [26] Ding, 2014 [27]	Moderate High
	2%	NR	Lee, 2006 [28] Burin, 2017 [29]	Moderate Low
		1 day	Bouvard, 2014 [30]	Moderate
		2 days	Zhang, 2018 [31]	Low
		3 days	Salamanna, 2018 [32]	Low
		12 days	Ciapetti, 2016 [33]	High
		14 days	Tsang, 2013 [34]	Low
		21 days	Tsang, 2013 [34]	Low
	5%	3 days	Gu, 2016 [35]	Moderate
		14 days	Ding, 2014 [27]	High
		49 days	Sengupta, 2010 [36]	Low
**➔** RUNX2-g **➔** ALP-g **➔** ALP **➔** ALP-activity **➔** Col1A1-g **➔** Col1A1 **➔** Osteocalcin **➔** Osteopontin-g **➔** Osteopontin **➔** Calcium deposit	1%	14 days	Jin, 2010 [37]	Low
		21 days	Binder, 2015 [38] Ding, 2014 [27]	Moderate High
	2%	5 days	Xu, 2007 [39]	Moderate
		8 days	Xu, 2007 [39]	Moderate
		12 days	Ciapetti, 2016 [33]	High
		14 days	Tsang, 2013 [34] Zhang, 2017 [31]	Low Moderate
		21 days	Tsang, 2013 [34] Lee, 2012 [40]	Low Moderate
	3%	14 days	Holzwarth, 2010 [41]	Low
	5%	NR	Russo, 2014 [42]	Moderate
		3 days	Gu, 2016 [35]	Moderate
		21 days	Binder, 2015 [38]	Moderate
		49 days	Sengupta, 2010 [36]	Low
	7%	NR	Iacono, 2018 [43]	Low
**↓** RUNX2-g **↓** RUNX22 **↓** ALP-g **↓** ALP **↓** ALP-activity **↓** Col1A1-g **↓** Col1A1 **↓** Osteocalcin-g **↓** Osteocalcin **↓** Osteopontin-g **↓** Osteopontin **↓** Calcium deposit	1%	NR	Lee, 2015 [44] Hsu, 2013 [45] Park, 2013 [46]	Moderate Low Moderate
		2 days	Ma, 2014 [47]	Moderate
		21 days	Ding, 2014 [27] Yao, 2017 [48] Xu, 2013 [49] Yang, 2011 [50] Cicione, 2013 [51]	High Low Moderate Low Moderate
	2%	NR	Burian, 2017 [29]	Low
		3 days	Salamanna, 2018 [32]	Low
		5 days	Xu, 2007 [39] Huang, 2012 [23]	Moderate High
		6 days	Pattappa, 2013 [52]	Moderate
		7 days	Zham, 2008 [53]	Moderate
		8 days	Xu, 2007 [39]	High
		14 days	Zhang, 2017 [31]	Moderate
		21 days	Huang, 2012 [23] Tsang, 2013 [34] Malladi, 2006 [54] Lee, 2012 [40]	Moderate Low Low Moderate
	3%	14 days	Holzwarth, 2010 [41]	Low
	5%	NR	Russo, 2014 [42]	Moderate
		6 days	Pattappa, 2013 [52]	High
		14 days	Inagaki, 2017 [55]	High
		21 days	Hopper, 2015 [56]	Low
		28 days	Merceron, 2010 [57]	High
**Cyclic Exposure**				
**➔** ALP activity **➔** Calcium deposit ****↓**** ALP **↓** RUNX2 **↓** Osteocalcin	1% 3% 5% 10%	15 days 2 × 3 min/day	Dìppolito, 2006 [58]	Moderate

NR: not reported; -g: genetic expression; RUNX2: runt-related transcription factor 2; ALP: alkaline phosphatase; Col1A1: collagen type 1 alpha1. High confidence in the association between exposure to the substance and the outcome: The true effect is highly likely to be reflected in the apparent relationship. Moderate confidence in the association between exposure to the substance and the outcome: The true effect may be reflected in the apparent relationship. Low confidence in the association between exposure to the substance and the outcome: The true effect may be different from the apparent relationship. Very low confidence in the association between exposure to the substance and the outcome: The true effect is highly likely to be different from the apparent relationship.

**Table 2:** Experimental details of in vivo studies included in this review

Hypoxia Effects on Outcomes	Sample				Intervention			References	Confidence Rating
	Type	Age	Size	Conditions (*n*)	Hypoxia Level (Meters; % PiO_2_)	Duration, Frequency	Exposure Type		
**Sustained Exposure**									
**↓**BV/TV **↓**Tb.N **↓**BMD-total	Sprague-Dawley rats	12 week-old		Hypoxia (*n* = 4) Normoxia (*n* = 4)	6000 m	3 weeks	Normobaric	Wang, 2017 [59]	High
**↓**BMD-spine	Healthy adults	24–58 years	5	NR	2500 m	24 weeks	Hypobaric	O´Brien, 2019 [60]	Moderate
**↓**SOS-R **➔**SOS-T **↓**SOS-M **↓**SOS-P **↓**T-score-R **↓**T-score-P **↑**ALP **↓**BAP **↑**Calcium deposit **↓**25-Vit D **↓**i-PTH **↓**CICP **↓**NTX **↓**DPD/Cr	Healthy adults	21–47 years	2600	Normoxia (*n* = 1300) Hypoxia (*n* = 1300)	3450 m	16 weeks	Hypobaric	Basu, 2014 [61]	High
**➔**SOS-R **➔**SOS-T **↓**SOS-M **↓**SOS-P **➔**Z-score-R **➔**Z-score-T **↓**Z-score-M **↓**Z-score-P **➔**Calcium **↑**Phosphorous **↓**ALP **↓**BAP **↓**25-Vit D **↓**Calcitonin **↑**i-PTH **➔**DPD/Cr	Healthy adults	21–47 years	221	Hypoxia (*n* = 221)	3000–3754 m (24 weeks) + 5400–6700 m (16 weeks)	40 weeks	Hypobaric	Basu, 2013 [62]	High
**Cyclic Exposure**									
**➔**BV/TV **➔**Tb.N **➔**BMD-total **➔**BMC-total **↓** BV/TV **↓**Tb.N **↓**BMD-total **↓**BMC-total	Sprague-Dawley rats	12 week-old	37	Hypoxia (*n* = 7) Normoxia (*n* = 6) Ovariectomized Hypoxia (*n* = 12) Ovariectomized Normoxia (*n* = 12)	3000–5000 m	2 weeks, 4 h/day	Normobaric	Wang, 2016 [63]	High
**↑**BMD-spine	Wistar albino rats	6 months-old	20	Hypoxia (*n* = 10) Normoxia (*n* = 10)	4500 m	5 weeks 5 days/week 5 h/day	Hypobaric	Guner, 2013 [64]	Moderate
**↑**BMD-total	Healthy adults	24.6 ± 2.8 years	28	Hypoxia (*n* = 15) Normoxia (*n* = 13)	15% PiO_2_	8 weeks 2days/week	Normobaric	Martínez-Guardado, 2019 [65]	High
**➔**BMD-total	Trained triathletes	27 years	18	Hypoxia Training (*n* = 9) Control (*n* = 9)	15% PiO_2_	7 weeks, 2days/week 60 min/day	Normobaric	Ramos-Campos, 2015 [66]	High
**↓**BMC-total **↑**BMC-total	Healthy young	26.4 years	14	Hypoxia Bed Rest (*n* = 14) Hypoxia Ambulatory (*n* = 14) Normoxia Bed Rest (*n* = 14)	4000 m	21 days	Normobaric	Rittweger, 2016 [67]	High
**Intermittent Exposure**									
**➔**BMD-spine	Menopausal Women with OSAS	56.3 ± 6.2 years	1201	NR	NR	NR	NR	Tng, 2018 [68]	High
**↑**CTX	Adults with OSAS	51 years	50	OSA (*n* = 30) Control (*n* = 20)	NR	NR	NR	Terzi, 2016 [69]	High
**↑**BMD-total	Adults with OSAS	68.6 ± 0.8 years	833	OSA (*n* = 459) Control (*n* = 373)	NR	NR	NR	Sforza, 2013 [70]	High
**➔**CTX **➔**RANKL **➔**OPG **➔**CTX **➔**RANKL **➔**OPG **↑**CTX **↓**RANKL **➔**OPG	Adults with OSAS	51.0 ± 13 years	65	Mild OSAS (*n* = 10) Moderate OSAS (*n* = 12) Severe OSAS (*n* = 28) Control (*n* = 15)	NR	NR	NR	Tomiyama, 2008 [71]	High

NR: not report; OSAS: obstructive sleep apnea syndrome; SOS-R: speed of sound at one-third of distal radius; SOS-P: speed of sound at the proximal third phalanx; SOS-M: speed of sound at fifth metatarsal; SOS-T: speed of sound at the mid-shaft tibia; BAP: bone specific alkaline phosphatase; 25-Vit D: 25-Hydroxy vitamin D3; i-PTH: intact parathyroid hormone; CICP: C-terminal propeptide of type I collagen; NTX: N-telopeptide of type I collagen; DPD/Cr: urinary DPD, creatinine ratio; BV/TV: bone volume; Tb.N: trabecular number; BMD- total: bone mineral density total; BMD- spine: bone mineral density dorsal spine; BMC-total: bone mineral content total; CTX: carboxy-terminal collagen cross-links; RANKL: Receptor activator for Nuclear Factor κ B Ligand; OPG: osteoprotegerin. High confidence in the association between exposure to the substance and the outcome: The true effect is highly likely to be reflected in the apparent relationship. Moderate confidence in the association between exposure to the substance and the outcome: The true effect may be reflected in the apparent relationship. Low confidence in the association between exposure to the substance and the outcome: The true effect may be different from the apparent relationship. Very low confidence in the association between exposure to the substance and the outcome: The true effect is highly likely to be different from the apparent relationship.

## Appendix A

### Appendix A.1. Search Strategies

PubMed:

((((((hypoxi*[Title] OR altitude[Title] OR “oxygen deprivation”[Title])) NOT growth*[Title]) NOT (tumour[Title/Abstract] OR cancer[Title/Abstract] OR pulmonary[Title/Abstract])) AND (“bone remodeling” OR “bone metabolism” OR osteog* OR “bone tissue remodeling” OR “bone mineral density”)) AND (“1900”[Date—Publication]: “3000”[Date—Publication])) AND English[Language]

Web of Science:

Title: (hypoxi* OR altitude OR “oxygen deprivation”) NOT Title:(growth*) NOT Topic: (tumour OR cancer OR pulmonary) AND Topic:(“bone remodeling” OR “bone metabolism” OR osteog* OR “bone tissue remodeling” OR “bone mineral density”)

Period of time: 1900–2019.

Language of search = English

## Appendix B

**Table A1:** Risk of bias and confidence rating for in vitro studies.

Reference	Risk of Bias Questions											Confidence Rating
	1	2	3	4	5	6	7	8	9	10	11	
Bouvard, 2014	-	-	NA	NA	+	-	-	+	+	++	+	Moderate
Binder, 2015	-	-	NA	NA	+	-	+	+	+	++	+	Moderate
Burian, 2017	-	-	NA	NA	+	-	-	+	+	+	+	Low
Ciappeti, 2016	++	-	NA	NA	+	-	-	+	-	++	+	High
Cicione, 2013	-	-	NA	NA	+	-	-	+	+	++	+	Moderate
D’Ippolito, 2006	-	-	NA	NA	+	-	-	+	+	++	+	Moderate
Deschepper, 2011	-	-	NA	NA	-	-	-	-	+	++	+	Moderate
Ding, 2014	-	-	NA	NA	++	-	-	++	+	++	+	High
Gao, 2013	-	-	NA	NA	+	-	-	+	+	++	+	Moderate
Gu, 2016	-	-	NA	NA	-	-	-	+	+	++	+	Moderate
Holwarth, 2010	-	-	NA	NA	+	-	+	+	+	+	+	Low
Hopper, 2015	+	-	NA	NA	+	-	-	-	+	+	+	Low
Hsu, 2013	-	-	NA	NA	-	-	-	-	+	+	+	Low
Huang, 2011	-	-	NA	NA	+	-	-	-	+	+	+	Low
Huang, 2012	-	-	NA	NA	+	-	-	+	+	++	+	Moderate
Iacono, 2018	-	-	NA	NA	+	-	-	+	+	+	+	Low
Inagaki, 2017	-	-	NA	NA	+	++	-	+	+	++	+	High
Jin, 2010	-	-	NA	NA	+	-	-	+	-	+	+	Low
Kalinina, 2015	-	-	NA	NA	+	-	-	+	+	++	+	Moderate
Lee, 2006	-	-	NA	NA	+	-	-	+	+	++	+	Moderate
Lee, 2012	-	-	NA	NA	+	-	-	+	+	++	+	Moderate
Lee, 2015	-	-	NA	NA	+	-	-	+	+	++	+	Moderate
Ma, 2014	-	-	NA	NA	+	-	-	+	+	++	+	Moderate
Malladi, 2006	-	-	NA	NA	+	-	-	+	+	+	+	Low
Merceron, 2010	-	-	NA	NA	++	-	-	++	+	+	+	High
Park, 2013	-	-	NA	NA	++	-	-	+	+	+	+	Moderate
Pattapa, 2013	++	-	NA	NA	++	-	-	+	+	+	+	High
Russo, 2013	-	-	NA	NA	+	-	-	++	-	+	+	Moderate
Salamanna, 2018	-	-	NA	NA	+	-	-	+	-	+	+	Low
Sengupta, 2010	-	-	NA	NA	+	-	-	+	+	+	+	Low
Tsang, 2013	-	-	NA	NA	+	-	+	+	+	+	+	Low
Wang, 2012	-	-	NA	NA	+	-	-	+	+	+	+	Low
Xu, 2007	-	-	NA	NA	++	-	-	+	+	+	+	Moderate
Xu, 2013	-	-	NA	NA	++	-	-	+	+	+	+	Moderate
Yang, 2011	-	-	NA	NA	+	-	-	+	+	+	+	Low
Yao, 2017	+	-	NA	NA	+	-	-	+	+	+	+	Low
Zham, 2008	-	-	NA	NA	+	-	-	++	+	++	+	Moderate
Zhang, 2017	-	-	NA	NA	++	-	-	+	+	+	+	Moderate
Zhang, 2018	-	-	NA	NA	-	-	-	+	-	+	+	Low

++: definitely low; +: probably low; -: probably high (not report); --: definitely high; NA: not applicable. ++ ++ High confidence in the association between exposure to the substance and the outcome. The true effect is highly likely to be reflected in the apparent relationship; + ++ Moderate confidence in the association between exposure to the substance and the outcome. The true effect may be reflected in the apparent relationship; ++ Low confidence in the association between exposure to the substance and the outcome. The true effect may be different from the apparent relationship; + Very low confidence in the association between exposure to the substance and the outcome. The true effect is highly likely to be different from the apparent relationship.

## Appendix C

**Table A2:** Risk of bias and confidence rating ratings for in vivo studies.

Reference	Risk of Bias Questions											Type of Study	Confidence Rating
	1	2	3	4	5	6	7	8	9	10	11		
Wang, 2016	+	-	NA	NA	-	-	++	+	++	++	+	EA	High
Wang, 2017	++	-	NA	NA	+	-	-	+	+	++	+	EA	High
Guner, 2013	-	-	NA	NA	+	-	+	+	-	++	+	EA	Moderate
Tomiyama, 2008	NA	NA	+	++	NA	NA	-	-	+	++	+	HCr-Se	High
Basu, 2013	NA	NA	++	+	NA	NA	+	+	-	++	+	HCr-Se	High
Basu 2014	NA	NA	++	+	NA	NA	+	+	-	++	+	HCo	High
Sforza, 2013	NA	NA	+	++	NA	NA	-	+	++	++	+	HCo	High
Terzi, 2015	NA	NA	--	++	NA	NA	-	++	++	++	+	HCo	High
Tng, 2008	NA	NA	--	++	NA	NA	-	+	++	++	+	HCo	High
O’Brien, 2018	-	-	NA	NA	NA	-	-	+	+	++	+	HCT	Moderate
Martínez-Guardado, 2019	++	++	NA	NA	NA	++	+	+	++	++	+	HCT	High
Ramos-Campos, 2015	+	-	NA	NA	NA	-	+	++	++	++	+	HCT	High
Rittweger, 2016	-	-	NA	NA	NA	-	++	++	+	++	+	HCT	High

++: definitely low; +: probably low; -: probably high (not report); --: definitely high; NA: not applicable; EA: experimental animal; HCT: human controlled trial; HCo: human cohort; HCr-Se: human cross-sectional. ++ ++ High confidence in the association between exposure to the substance and the outcome: The true effect is highly likely to be reflected in the apparent relationship; + ++ Moderate confidence in the association between exposure to the substance and the outcome: The true effect may be reflected in the apparent relationship; ++ Low confidence in the association between exposure to the substance and the outcome: The true effect may be different from the apparent relationship; + Very low confidence in the association between exposure to the substance and the outcome: The true effect is highly likely to be different from the apparent relationship.

## Appendix D

**Table A3:** Summary risk of bias domain assessment for animal, human and in vitro studies included in the review.

Domain	Animal Studies						Human Studies						In Vitro Studies					
Selection	**++**		**+**		**-**		**+**			**-**			**++**		**+**		**-**	
Performance	**+**			**-**			**-**						**++**		**+**		**-**	
Attrition/exclusion	**++**			**-**			**++**		**+**		**-**		**+**			**-**		
Detection	**++**			**+**			**++**		**+**		**-**		**++**		**+**		**-**	
Selective Reporting	**++**			**+**			**++**			**+**			**++**			**+**		

++: definitely low (dark green colour); +: probably low (light green colour); -: probably high (not report; red colour).

## Appendix E

**Table A4:** Confidence rating for a health effect given strengths and weaknesses of a collection of animal and human studies.

Type of Study	Level of Confidence for Health Effect Bone Remodelling
Experimental Animal	High
Human Controlled Trial	High
Human Cohort	High
Human Cross-Sectional	High
In Vitro Studies	Moderate
